# Genome sequence of *Plasmopara viticola* and insight into the pathogenic mechanism

**DOI:** 10.1038/srep46553

**Published:** 2017-04-18

**Authors:** Ling Yin, Yunhe An, Junjie Qu, Xinlong Li, Yali Zhang, Ian Dry, Huijuan Wu, Jiang Lu

**Affiliations:** 1 College of Food Science and Nutritional Engineering, China Agricultural University, Beijing 100083, China; 2 Guangxi Crop Genetic Improvement and Biotechnology Laboratory, Guangxi Academy of Agricultural Sciences, Nanning 530007, China; 3 Beijing Center for Physical and Chemical Analysis, Beijing 100089, China; 4 CSIRO Agriculture & Food, Wine Innovation West Building, Hartley Grove, Urrbrae, SA 5064, Australia; 5 Center for Viticulture and Enology, School of Agriculture and Biology, Shanghai Jiao Tong University, Shanghai 200024, China

## Abstract

*Plasmopara viticola* causes downy mildew disease of grapevine which is one of the most devastating diseases of viticulture worldwide. Here we report a 101.3 Mb whole genome sequence of *P. viticola* isolate ‘JL-7-2’ obtained by a combination of Illumina and PacBio sequencing technologies. The *P. viticola* genome contains 17,014 putative protein-coding genes and has ~26% repetitive sequences. A total of 1,301 putative secreted proteins, including 100 putative RXLR effectors and 90 CRN effectors were identified in this genome. In the secretome, 261 potential pathogenicity genes and 95 carbohydrate-active enzymes were predicted. Transcriptional analysis revealed that most of the RXLR effectors, pathogenicity genes and carbohydrate-active enzymes were significantly up-regulated during infection. Comparative genomic analysis revealed that *P. viticola* evolved independently from the Arabidopsis downy mildew pathogen *Hyaloperonospora arabidopsidis*. The availability of the *P. viticola* genome provides a valuable resource not only for comparative genomic analysis and evolutionary studies among oomycetes, but also enhance our knowledge on the mechanism of interactions between this biotrophic pathogen and its host.

Oomycetes, which include a large number of notorious plant pathogens, are phylogenetically related to diatoms and brown algae in the Stramenopiles^1^. *Plasmopara viticola* (Berk. & M. A. Curtis) Berl. & De Toni is the causal agent of grapevine downy mildew, a destructive oomycete disease of viticulture worldwide^2^. *Plasmopara viticola* is a strictly obligate biotrophic organism since its survival depends on living host cells and cannot be propagated on artificial media^3^. This pathogen is native to North America and was accidentally introduced into Europe via infected cuttings at the end of the 19^th^ century^4^. However, recent research^5^,^6^ has demonstrated the existence of a complex of cryptic *P. viticola* species specialized on different wild *Vitis* sp. and cultivated grapevines. The *P. viticola* strains collected in China were also found quite distinct from these collected in North America and Europe^7^,^8^ although more studies and large sample sizes are necessary.

All major *Vitis vinifera* cultivars are highly susceptible to *P. viticola*. In the absence of effective chemical protection, downy mildew causes severe damage to grapevine leaves and bunches and may lead to complete loss of the crop. Multiple fungicide applications may be required during the growing season, but this is costly for the grower and has a negative impact on the environment. Meanwhile, long-term fungicide application can lead to resistance risk^9^. In order to develop more sustainable production systems for viticulture, new strategies of protection against this pathogen are needed through the development of resistant grapevine germplasm. So it is necessary to identify undiscovered genetic resources^10^,^11^,^12^,^13^,^14^, to diversify the combination of resistance genes currently present in different breeding lineages^15^ or to use combination of control methods to overcome the partial host resistance^16^,^17^.

Next-generation sequencing technology and bioinformatics analysis packages have greatly facilitated studies on the genomes and transcriptomes of plant pathogens, including oomycetes. In the past decade, genome sequences have been published for several oomycete species including the biotrophic downy mildews *Hyaloperonospora arabidopsidis*^18^, *Plasmopara halstedii*^19^ and *Pseudoperonospora cubensis*^20^, the white rusts *Albugo candida*^21^ and *Albugo laibachii*^22^, the hemibiotrophic *Phytophthora* species *Phytophthora ramorum* and *Phytophthora sojae*^23^, *Phytophthora infestans*^24^, *Phytophthora capsici*^25^, *Phytophthora lateralis*^26^, and the necrotrophic phytopathogen *Pythium ultimum*^27^. Recently, a draft genome sequence of *P. viticola* isolate INRA-PV221 collected in Bordeaux, France has also been released^28^. The availability of these genome sequences greatly facilitates studies on the interaction between oomycete pathogens and their host, and, in particular, the interaction between pathogen effectors and host proteins involved in resistance pathways. Previous genome-wide analysis has revealed the presence of hundreds of genes within the genomes of these pathogens that encode secreted proteins that could potentially act as effectors^18^,^19^,^20^,^21^,^22^,^23^,^24^,^25^,^26^,^27^. The two major classes of secreted effectors that have been identified in the oomycete genomes are the RXLR and CRN(crinkling and necrosis-inducing) effectors^29^. The RXLR effectors contain a conserved N-terminal amino acid motif consisting of arginine, any amino acid, leucine and arginine whereas the CRN effectors feature a conserved N-terminal LXLFLAK motif connected to diverse C-terminal effector domains. Functional studies have demonstrated that suppression of host immunity is a major function of both RXLR and CRN effectors^30^,^31^. In modern resistance breeding, effectors are emerging as tools to accelerate and improve the identification, functional characterization, and deployment of resistance genes^32^.

Parasitism of plants evolved at least twice independently in the *Peronosporalean* lineage. Within this lineage, obligate biotrophy evolved independently in white blister rusts and downy mildews^1^. A multi-gene phylogenetic analysis of downy mildews based on selected coding and non-coding nuclear and mitochondrial loci revealed that *Plasmopara* and *Hyaloperonospora* are positioned in different clades^33^. The genomes of four oomycetes that cause downy mildew disease have previously been reported (*H. arabidopsidis, P. halstedii, P. cubensis and P. viticola*). Here we report the genome sequence of a Chinese *P. viticola* isolate ‘JL-7-2’, which was originally collected from infected leaves of a ‘Beta’ grapevine (*V. riparia* × *V. labrusca*) growing in northeastern China. In this study, a novel strategy combining data obtained from both Illumina/Solexa sequencing technology and the Pacific Biosciences (PacBio) sequencing platform has been used. The availability of this genome sequence not only provides insight into the pathogenicity mechanism of this pathogen but also presents valuable additional information for further understanding the evolution of downy mildews.

## Results

### Genome assembly and quality assessment

The genome of *P. viticola* isolate ‘JL-7-2’ was sequenced using a combination of Illumina and PacBio RS technologies. The *P. viticola* isolate ‘JL-7-2’ was selected for genome sequencing because it is the most virulent strain, based on pathogenicity analysis, of the grapevine downy mildew isolates collected throughout the Chinese continent^8^. Illumina paired-end libraries of 180 bp, 500 bp, 800 bp and 1,000 bp were constructed and sequenced to 138× coverage. In addition, two mate-pair libraries of 3 kb and 6 kb were constructed and sequenced at 31× coverage to build super contigs (Supplementary Table S1). The PacBio RS sequencing produced 38× coverage with an average length of 4,966 bp. PacBio long reads were integrated with the *de novo* assembly of Illumina sequences, to fill in gaps and join scaffolds, with PBJelly2. The assembly combining Illumina data and PacBio data produced a better overall result than using Illumina data alone (Supplementary Table S2). The final assembly resulted in 2,165 scaffolds, spanning 101.3 Mb. This is consistent with the previous estimates of the *P. viticola* genome size of 113.55 ± 6.68 and 118.44 ± 7.53 Mb based on Feulgen staining analysis of an isolate collected from Australia^34^. Over 50% of the genome assembly was covered by 172 scaffolds with an N50 scaffold length of 172.3 kb (Table 1) with the largest scaffold 806 kb in length. To further assess the quality of this genome assembly, the N length versus the N number (number of contigs in each N category) from N10 to N100 was plotted and this indicated that 90% of the assembled genome was covered by 714 scaffolds, whilst the remaining 10% of the genome assembly is highly fragmented in 1,456 scaffolds (Supplementary Fig. S1). The results of the genome assembly of *P. viticola* isolate ‘JL-7-2’ are in close agreement with the recently published genome assembly statistics of *P. viticola* isolate INRA-PV221^28^ (Supplementary Table S3).

The CEGMA and BUSCO methods were used to estimate the degree of completeness of the assembled gene space. Most of the gene space was covered, as 234 complete and 8 partial models of 248 CEGs were identified within the *P. viticola* draft genome (Supplementary Fig. S2). Of the 429 conserved eukaryotic proteins provided by BUSCO^35^, 362 were found to be present in the genome (Supplementary Table S4), suggesting this assembly covers most of the genes of *P. viticola*. This was further supported by the RNA-Seq data. More specifically, 92.3% of the transcripts assembled from our RNA-Seq data of different isolates and 94.1% of 427 validated *P. viticola* genes encoding proteins in the UniProt database (release 2014_07) could be found in this assembly. All these assessments indicate that the genome assembly is of high quality and encompasses a high coverage of the *P. viticola* coding regions.

### Prediction of coding regions in the *P. viticola* genome

A total of 17,014 protein-coding gene models were predicted from the *P. viticola* genome assembly. Using RNA-Seq analysis, we were able to find evidence of expression of at least 11,670 (68.2%) of these predicted genes in at least one of the isolates at some stage during the infection process (Supplementary Table S5). This is similar to the predicted number of genes in *P. infestans*^24^ (240 Mb, 17,887 genes), but lower than *P. sojae*^23^ (95 Mb, 19,027 genes) and higher than *H. arabidopsidis*^18^ (100 Mb,14,543 genes) and *P. ramorum*^23^ (65 Mb, 15,743 genes). The average gene length is approximately 1,344 nt with a mean of 2.4 exons per gene and an average exon length of 483 nt. A total of 90.5% of these gene models had at least one match (E-value 1e-5) in the public protein databases (NCBI’s non-redundant protein databases, GO, SWISS-PROT databases). Conserved protein domains were identified in 11,987 and 8,940 of the predicted genes by using InterPro and Pfam programs, respectively. Products of 2,378 gene models were associated with 380 KEGG (Kyoto Encyclopedia of Genes and Genomes) pathway maps.

### Annotation of repeat sequences

The assembled genome has an overall GC content of approximately 45% which increases to 50% within the coding regions. Almost 26% of the assembled *P. viticola* genome assembly consists of repetitive elements. The majority of these repetitive elements are transposable elements (TEs) of which the long terminal repeat (LTR) elements Gypsy and Copia, are the predominant class (~14%) (Supplementary Table S6). DNA transposons and non-LTR retrotransposons (LINEs and SINEs) each represent approximately 3% of the genome, but show considerable diversity. The percentage of repetitive sequences within the *P. viticola* genome is comparable to that found in the genomes of a number of other oomycete pathogens including *P. ramorum*^23^ (28%), *A. laibachii*^22^ (22%), *P. capsici*^25^ (19%) and *P. tabacina*^36^ (24%), even though their genome sizes are smaller than that of *P. viticola*. However, it is significantly lower than that previously found in the genomes of *H. arabidopsidis*^18^ (43% repeats in an estimated 100 Mb genome) and *P. infestans*^24^ (74% of the 240 Mb genome).

### Identification of putative secreted proteins

**Figure 1:**
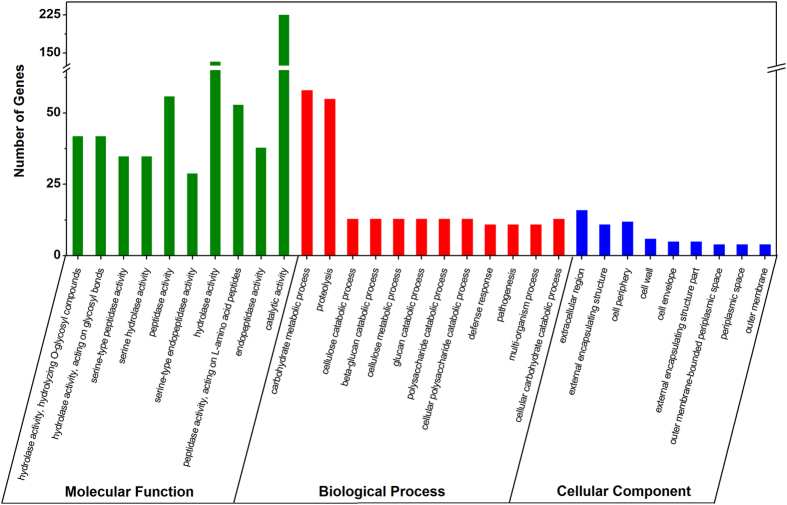
GO enrichment analysis of predicted secreted proteins encoded in the *P. viticola* genome.

The proteins secreted by plant pathogenic fungi and oomycetes, particularly the effector secretomes, are essential for successful infection via manipulation of host cell structure and function^37^,^38^. Of the 17,014 predicted gene models in the *P. viticola* genome assembly, 1,301 were predicted to be secreted based on signal peptide prediction and an absence of transmembrane domains. GO terms were assigned to a total of 652 of the 1,301 candidate secreted proteins across the three major categories: molecular functions (314), biological processes (260) and cellular components (78) (Fig. 1). Within the molecular function class, proteins with hydrolase and peptidase activity were highly represented. Within the biological process category, proteins involved in carbohydrate metabolism were the most abundant, while in the cellular component category, proteins associated with the extracellular region, external encapsulating structure and cell periphery were the most highly represented.

### Identification of candidate PvRXLR effectors

**Figure 2:**
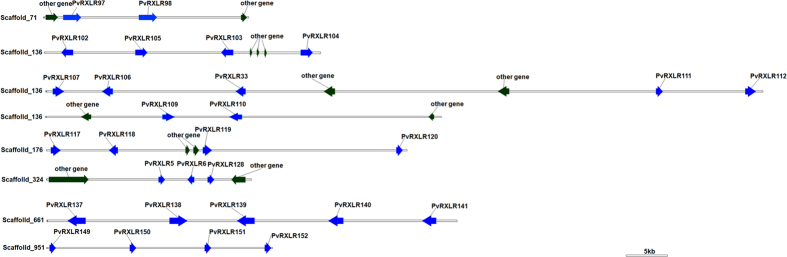
Clusters of *PvRXLR* genes in the *P. viticola* genome. Blue arrows represent putative PvRXLR effector genes while dark green arrows indicate other genes.

**Figure 3:**
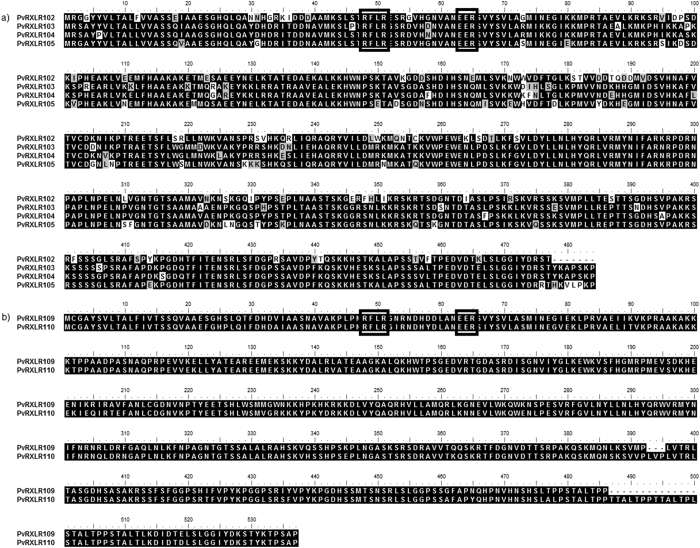
Alignment of translation products of predicted PvRXLR genes found in Scaffold 136. The RXLR and dEER motifs are indicated with a black boxes. The alignment was constructed using BioEdit3.3.19.0 software. The threshold (%) for shading was set at 50. Similar amino acid residues are shaded grey and identical amino acid residues are shaded black.

RXLR effectors are the major class of cytoplasmic effectors secreted by oomycete pathogens. The presence of the conserved RXLR motif makes it feasible to identify candidate RXLR effector genes within the genome sequence of oomycete pathogens^39^. A total of 100 putative RXLR-dEER like effectors (Supplementary Table S7) were predicted from the secretome of the Chinese *P. viticola* isolate ‘JL-7-2’, and 31 of which had previously been identified by *de novo* assembly of transcriptome data from three *P. viticola* isolates infecting on grapevine leaves^40^. Mestre *et al*.^41^ also recently reported the identification of 44 putative RXLR effectors from transcriptome sequencing of a European *P. viticola* isolate. However, only 18 of these PvRXLRs from the European isolate were common in comparison to the 100 PvRXLRs identified from the Chinese isolate. Over 50% of the genes predicted to encode PvRXLRs were clustered in a small region of the *P. viticola* genome with a length of 2.28 Mb (Fig. 2). For example, one scaffold of 568 kb (Scaffold_136) and another scaffold of 322 kb (Scaffold_71) contained 16 and 8 PvRXLR genes, respectively (Fig. 2). Interestingly, the PvRXLRs are predominantly located in genomic regions that contain relatively few genes and a high frequency of LTR transposons, DNA transposons and other repetitive elements (Supplementary Table S7). Sequence alignments of the clustered PvRXLRs revealed that multiple, near-identical copies of RXLR effector genes are present in *P. viticola* genome (Fig. 3). Previous studies reported that this phenomenon was prevalent in oomycete genomes^41^,^42^,^43^.

The genome of arabidopsis downy mildew (*H. arabidopsidis*) was predicted to encode 134 RXLR-like effectors^18^. It appears that both grapevine and arabidopsis downy mildew contain significantly less RXLR effectors than those in *Phytophthora* species in which 350–563 were predicted^18^,^23^,^24^. The PvRXLRs show only limited similarity to RXLR effectors identified from *H. arabidopsidis, P. infestans* and *P. sojae* as only 18 of 100 PvRXLRs showed more than 30% amino acid sequence identity to RXLRs from the other oomycete species. Exceptions to this are PvRXLR13 and PvRXLR129 which are highly conserved across *P. viticola, P. halstedii, H. arabidopsidis, P. infestans* and *P. sojae* with more than 70% sequence identity (Supplementary Fig. S3).

### Identification of CRN effectors

In addition to RXLR effectors, oomycete pathogens also produce a large number of CRN effector proteins. The CRN protein family encodes modular proteins which are characterized by a conserved N-terminal LXLFLAK motif, a recombination site motif HVLVVVP (DWL domain) and diverse C-terminal effector domains^24^,^44^,^45^. However, previous analysis of oomycete CRN proteins has shown that CRN signal peptides are not always readily detected by SignalP analysis^27^,^45^. Using the mining method described by Yin *et al*.^40^, a total of 90 putative PvCRN proteins were identified from the *P. viticola* genome (Supplementary Table S9). Of these, only 26 were predicted to have signal peptide based on signalP3.0-HMM or the Phobius prediction method^46^. Sequence comparison revealed that only four of the predicted CRN sequences found in the ‘JL-7-2’ isolate were common with the CRN effectors previously identified in the SL and SC *P. viticola* isolates^41^. Furthermore, there was little overall homology between the predicted CRN effector proteins from *P. viticola* and those present in other oomycete species. Indeed, only one predicted PvCRN (PvCRN70) was found to contain a region that is highly conserved in CRN proteins from all 4 oomycete genomes examined (Supplementary Fig. S3). However, a HMM model search found that 20 of the 36 domain structures previously defined for the *Phytophthora* CRN effector repertoire^24^ are also present in the C-terminal domain of the PvCRN proteins, the most common of which are the DXX and DXZ effector domains (Supplementary Table S8).

### Predicted pathogenicity genes in the *P. viticola* secretome

In addition to the large number of RXLR and CRN proteins, which are the two major classes of secreted effectors, a number of other families of secreted proteins were predicted to be encoded in the *P. viticola* genome including proteases, glycoside hydrolases, elicitins and elicitin-like proteins, and cell wall degrading enzymes including pectin esterases, pectin lyases and phospholipases. To identify genes encoding potential pathogenicity proteins in the *P. viticola* secretome, BLAST analyses were performed against the Pathogen-Host Interaction (PHI) Database. A total of 261 proteins were predicted to be involved in virulence and pathogenicity according to the PHI database (Supplementary Table S10) including 3 RXLR effectors and 10 CRNs listed in Supplementary Tables S7 and S9.

CAZymes have also been reported as pathogenicity factors in plant pathogens including oomycetes^47^. Putative CAZymes in *P. viticola* secretome were identified using the dbCAN database. Of the 1301 secreted proteins analysed, 95 were predicted as belonging to a CAZyme families. Moreover, 35/95 of the predicted CAZymes were present in the PHI database of the potential pathogenicity proteins including 15 families of glycoside hydrolases (GHs), 6 families of carbohydrate esterases (CEs), 6 families of carbohydrate-binding modules (CBMs), 3 families of glycosyl transferases (GTs), 4 families of auxiliary activities (AAs) and one polysaccharide lyases (PLs) (Supplementary Table S11). Among all the CAZyme families predicted in the *P. viticola* secretome, the GH family is the most highly represented (60 genes), followed by the CE proteins (16 genes). The GHs were also found to be the most abundant apoplastic effectors in our previous studies on *P. viticola* transcriptomics during infection^40^.

We have previously shown that inoculation of *V. amurensis* cv. Shuanghong leaf tissues with *P. viticola* isolates ‘JL-7-2’ and ‘ZJ-1-1’ resulted in an incompatible or compatible interaction^48^. Comparison of gene expression profiles of these two isolates at 12, 24, 48 and 72 h post inoculation of *V. amurensis* cv. Shuanghong showed that there were a significant difference in transcriptional responses between ‘JL-7-2’ and ‘ZJ-1-1’ over the time course of infection. Of particular note, 77% of the predicted RXLR effectors, 72.8% of the potential pathogenicity genes and 72.6% putative CAZymes are identified as up-regulated differently expressed genes in ‘ZJ-1-1’ (Supplementary Table S12). In addition, 28 out of 90 CRNs were also up regulated in ‘ZJ-1-1’.

### Comparisons with other oomycete genomes

**Figure 4:**
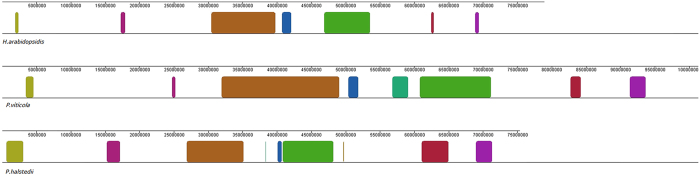
Synteny blocks shared between *P. viticola* and other sequenced downy mildew genomes including *P. halstedii* and *H. arabidopsidis*. Each coloured block represents a region in *P. viticola* that is colinear with a region of the other genomes.

When comparing *P. viticola* with other oomycete pathogens, only 35–57% of the predicted proteins in the *P. viticola* genome shared more than 60% amino-acid identity with those in *H. arabidopsidis*^18^, *P. infestans*^24^, *P. sojae*^23^ and *P. halstedii*^19^, respectively. More evidently, the percentage of proteins with >60% identity decreased significantly in secretome comparisons (Supplementary Table S13). These findings imply that secreted proteins of *P. viticola* may be subjected to more selection pressure and have evolved more rapidly than other proteins. OrthoMCL analysis found that the highest number of orthologous genes was observed between *P. viticola* and the downy mildew biotroph *P. halstedii* (9,567) (Table 2). Surprisingly, however, fewer orthologous genes were identified between *P. viticola* and the other biotrophic downy mildew pathogen *H. arabidopsidis* (6,718) than between *P. viticola* and the hemibiotrophic oomycete species *P. sojae* (8,164), and *P. infestans* (8,545). Furthermore, *P. viticola* showed a higher proportion of conserved proteins to *P. infestans* than to *H. arabidopsidis* (Supplementary Fig. S4a). Phylogenetic analyses based on a comparison of 3,249 one-to-one orthologues found that *P. viticola* and *P. halstedii* displayed a sister group relationship while *H. arabidopsidis* placed outside of the downy mildew group and *Phytophthora* clade (Supplementary Fig. S4b). In addition, the *P. viticola* genome exhibits larger areas of synteny to *P. halstedii* in comparison to *H. arabidopsidis* (Fig. 4).

It has been reported that obligate biotrophic oomycete lost some metabolic pathways^18^,^22^,^49^. Intriguingly, comparative genome analysis and genomic PCR revealed that *P. viticola* still retains the genes encoding a nitrate reductase, a nitrate transporter and a sulphite oxidase (Supplementary Tables S14 and S15) and they are all expressed during infection (Supplementary Table S5). These genes are also present in *P. halstedii*, but appear to be missing in the genomes of other biotrophic oomycetes *H. arabidopsis*^18^ and *A. laibachii*^22^ (Supplementary Table S14). Similar to other haustorium-forming oomycetes, *P. viticola* also lacks the thiamine-phosphate synthase gene, but retains the thiamine pyrophosphokinase gene which encodes key enzymes in thiamine biosynthetic pathway.

## Discussion

Here, we present a high-quality draft genome of the economically important grapevine pathogen, *P. viticola*, assembled with a combination of data derived from Illumina/Solexa and PacBio/Smart sequencing methodologies. The so-called “third generation” single-molecule sequencing technology developed by Pacific Biosciences (PacBio) has been widely used in recent years because it generates much longer reads and does not require a PCR step in sample preparation^50^. In this study, an improved genome assembly was obtained after the PacBio long reads were used for gap filling and scaffolding of Illumina generated sequence. The length of the largest scaffold was increased from 692.6 kb to 805.7 kb and the percentage of gaps was decreased to 16.71% from 27.94%. Furthermore, our analysis indicates that most of the gaps that were closed by the PacBio reads were located in repeat-rich regions, demonstrating that PacBio sequencing is a powerful tool to break through the bottlenecks often encountered during the of assembly of large repetitive regions. The advantages of including longer PacBio reads, e.g. hybrid assembly or PacBio reads combined with optical mapping, were also demonstrated by previous studies in the sequencing of bacterial and fungal genomes^51^,^52^. Especially for small genome, near-gapless even completely finished genome could be obtained^53^,^54^.

In plant-pathogen interactions, secreted proteins play an important role during early colonization and pathogenesis. GO analysis of the predicted PvRXLR secretome revealed that majority of secreted proteins displayed activities associated with proteolysis and hydrolysis, in particular glycoside hydrolases. Although the number of the CAZymes in *P. viticola* secretome is less than that in *Phytophthora* species^47^, GHs family are also the most abundant. It suggested that the degradation of the plant cell wall is an important step for the pathogen successful colonization.

In this study, we identified 100 PvRXLR effectors which were found to cluster in specific regions of the *P. viticola* genome. The majority of clusters contain two to six related genes, suggesting that local duplications might be involved in expansion of effectors in the *P. viticola* genome. Similarly, Burstein *et al*.^55^ reported that effectors cluster non-randomly in the genome of the bacterium *Legionella pneumophila*. Clustering of effectors has also been observed in the maize smut pathogen *Ustilago maydis*^56^ and rice false smut pathogen *Ustilaginoidea virens*^57^. Interestingly, plant *R* genes are also frequently found to occur in clusters^58^. The existence of gene-for-gene relationships between host resistance (R) and pathogen avirulence (AVR) genes is well established^59^. Thus, the clustering of both host *R* genes and pathogen effectors may reflect the importance of co-evolution between the host and pathogen. It has been reported that the majority of RXLR effectors in *Phytophthora* could manipulate host immunity^60^,^61^. The RXLR effectors encoded in the *P. viticola* genome also appear potentially able to contribute to virulence based on the transcriptional data and the ability of the majority of effectors examined to suppress PCD triggered by BAX or INF1 in *N. benthamiana*^40^,^62^.

CRN effectors are cytoplasmic effectors which were originally identified from *P. infestans* transcripts^63^. They have been identified in all plant pathogenic oomycetes sequenced to date. The genome of *P. viticola* is predicted to encode 90 CRN-like proteins, which is similar to the number of CRN effectors predicted to be present in the genomes of *H. arabidopsidis* and *P. halstedii*^18^,^19^. The predicted PvCRN sequences show a large amount of variation within the C-terminal region. Stam *et al*.^45^ reported that the DXX domain appears to have emerged early in oomycete evolution. Previous studies on *Phytophthora* CRN proteins have shown that they target the host nucleus and enhance pathogen virulence^29^,^64^,^65^,^66^. However, the function of CRNs in biotrophic oomycetes is yet to be determined.

Our analysis showed a high number of putative RXLR and CRN effectors, differing from those of the European isolates^41^. Different numbers of effectors were also predicted from the two Chinese isolates and one Australian isolate in our previous transcriptome study^40^. This phenomenon is the result of pathogen adaptation to different grapevine genotypes.

It has been reported that plant pathogens in the *Peronosporalean* lineage evolved at least twice independently^1^. When the genome sequence of *P. viticola* was compared with *H. arabidopsidis, P. halstedii*, and *Phytophthora* species, it was found to be most closely related to *P. halstedii*. This is consistent with a number of previous molecular phylogenetic analyses performed based on Dl-3 and D7-8 nrLSU rDNA^67^ and ITS2 sequence data^68^. However, *P. viticola* was also found to be more closely related to the *Phytophthora* species than to *H. arabidopsidis*. Unlike the *Phytophthora* species, all of the biotrophic oomycetes considered in this study appear to lack the nitrogen and sulfur metabolic pathways, as genes encoding both nitrite reductase and sulfite reductase are absent. However, both the *P. viticola* and *P. halstedii* genomes have retained genes encoding a nitrate reductase, a nitrate transporter and a sulphite oxidase. Furthermore, the detectable expression of these genes during grapevine downy mildew infection suggests that these genes still play important roles in biotrophic lifestyle of *P. viticola*. When considered together, these findings strongly suggest that *P. viticola* and *H. arabidopsidis* evolved biotrophy independently and *P. viticola* could be at a less advanced stage of evolution to biotrophy in comparison with *H. arabidopsidis* and *A. laibachii*. Phylogenomic analysis of *P. halstedii* also supports the independent evolution of two in the three major downy mildew lineages because downy mildews do not appear to be monophyletic^19^.

Like barley powdery mildew^49^ and flax rust^69^, *P. viticola* has lost genes encoding proteins of the thiamine biosynthetic pathway. Interestingly, all the five oomycetes (*P. viticola, P. infestans, P. sojae, H. arabidopsidis* and *A. laibachii*), appear to have lost these thiamine biosynthetic pathway genes but still retained the thiamine pyrophosphokinase gene that encodes thiamine phosphorylation. One explanation may be that thiamine may be easier to acquire from the host than the other nutrients. Therefore, in comparison with other metabolic pathways, the thiamine biosynthesis pathway may be the first to be lost during the evolutionary process to biotrophy. Thiamine diphosphate (ThDP) and thiamine triphosphate (ThTP), the two active forms of thiamine, can be formed in 1–2 steps from free thiamine using thiamin pyrophosphokinase, an enzyme that is encoded in the genomes of all biotrophic plant pathogens analyzed to date. Thus, we propose that biotrophic pathogens, such as the *P. viticola*, may be able to obtain thiamine directly from the plant host cells and then phosphorylate it to the ThDP and THTP using thiamin pyrophosphokinase. Under such circumstances, there is no longer a need to maintain genes of the thiamine biosynthetic pathway within the oomycete pathogen. In conclusion, the release of the *P. viticola* genome information is a valuable addition to the existing oomycete genome resources, which will provide useful information for genetic and evolutionary studies of these pathogens, as well as insights into genes involved in interactions with their hosts. Furthermore, our genome data and interpretation provides an unparalleled opportunity to address the molecular mechanisms of pathogenesis of *P. viticola* and evolution to the obligate biotrophic lifestyle.

## Materials and Methods

### P. viticola isolate collection and DNA preparation

The *P. viticola* isolates ‘JL-7-2’ and ‘ZJ-1-1’ was originally purified by single sporangiophore transfer from infected leaves of ‘Beta’ grapevine (*V. riparia* × *V. labrusca*) collected from Jilin province, China. The isolates were propagated by subsequent inoculations onto *V. vinifera* cv. Thompson Seedless plants grown under controlled greenhouse conditions (22 °C under a 16 h light/8 h dark cycle). The genomic DNA of isolate ‘JL-7-2’ that was used for genome sequencing was isolated from sporangia and sporangiophores, collected from infected leaves, using the CTAB method^70^.

### Illumina/Solexa sequencing

Six libraries with different insert sizes were constructed using two different methods. The small insert size libraries, including fragments of 180 bp, 500 bp, 800 bp and 1,000 bp, were constructed following the protocol outlined in of the TruSeq^TM^ DNA Sample Preparation v2 Guide. The 3 kb and 6 kb libraries were constructed according to the following different protocols for Nextera^®^ mate-pair sample preparation. The 3 kb library was constructed using the gel-free protocol and the 6 kb library used the gel-plus protocol. Sequencing was conducted on an Illumina Hiseq2000/2500 with a paired-end module generating reads of 100 bp.

### PacBio/SMRT single molecule sequencing

Genomic DNA samples were sheared to an average size of 10 kb via adaptive focused acoustics using a Covaris S220 focused-ultrasonicator (Covaris, MA, USA), end repaired and ligated to hairpin adapters. Incompletely formed SMRTbell templates were digested with a combination of Exonuclease III and Exonuclease VII. SMRT sequencing was carried out on the PacBio RS using standard protocols.

### RNA extraction, library construction and transcriptome sequencing

RNA samples for transcriptome analysis were isolated from downy mildew-infected grape discs at time points ranging from 12–96 h post inoculation as described previously^40^,^48^. Isolates ‘JL-7-2’, ‘ZJ-1-1’ and ‘CSIRO-L-2’ were inoculated onto discs or attached leaves of *V. vinifera* cv. Thompson seedless, *V. amurensis* cv. Shuanghong and *V. vinifera* cv. Cabernet Sauvignon respectively. Total RNA was extracted from infected leaves using a modified CTAB method^71^. RNA degradation and contamination was monitored on 1% agarose gels. RNA purity was checked using a NanoPhotometer^®^ spectrophotometer (IMPLEN, CA, USA). RNA concentration was measured using a Qubit^®^ RNA Assay Kit with a Qubit^®^ 2.0 Flurometer (Life Technologies, CA, USA). RNA integrity was assessed using the RNA 6000 Nano Assay Kit of the Bioanalyzer 2100 system (Agilent Technologies, CA, USA). All four RNA samples had RIN (RNA Integrity number) values greater than 8. A total of 3 μg RNA per sample was used as input material for the sample preparations. Sequencing libraries were generated using a NEB Next Ultra Directional RNA Library Prep Kit from Illumina (NEB, Ipswich, USA) according to the manufacturer’s instructions and four index codes were added to attribute sequences to each sample. The clustering of the index-coded samples was performed on a cBot Cluster Generation System using TruSeq PE Cluster Kit v3 -cBot-HS (Illumina, CA, USA) according to the manufacturer’s instructions. After cluster generation, the library preparations were sequenced on an IlluminaHiseq 2000 platform and 100 bp paired-end reads were generated.

### Genome assembly and quality assessment

Raw reads were trimmed using Cutadapt^72^ with the default parameters. FastQC was used to estimate the quality of trimmed reads^73^. The paired-end Illumina reads were assembled with ALLPATHS-LG software^74^ using default parameters. To improve the quality of the assembly, Illumina reads were also generated from three small libraries (180 bp, 500 bp and 800 bp) which were used to fill gaps using GapFiller^75^. Finally, PacBio long reads were used to fill gaps in the scaffolds using PBJelly2 software^76^. CEGMA^77^ and BUSCO (version 1.1b)^35^ pipelines were used to estimate the completeness and correctness of the genome assembly. BUSCO was run in ‘OGS’ (gene set/proteome) mode. Assembled transcripts from our previously published transcriptome data^40^ and protein sequences in the UniProt database were aligned to the *P. viticola* genome using BLAT and tBlastn. All the RNA-Seq reads were mapped to the *P. viticola* genome using TopHat v2.0.8b^78^. The number of unique reads mapped to each gene were counted using htseq-count fromHTSeq-0.6.1^79^. Genes with a read counts greater than or equal to 10 were considered expressed. Differential gene expression was analyzed using the NOISeq package version 2.6.0^80^ according to Li *et al*.^48^. This Whole Genome Shotgun project has been deposited at DDBJ/ENA/GenBank under the accession MTPI00000000. The version described in this paper is version MTPI01000000.

### Analysis of repeats

The *de novo* repeat family identifier and modeling package RepeatModeler (http://www.repeatmasker.org/RepeatModeler.html) was used to generate a repeat library database. This resulted in the creation of an extensive, uncurated library of putative *P. viticola* repeats. This database, along with RepBase, was searched to generate repeats using RepeatMasker^81^. RepeatModeler runs as a wrapper around three other de novo repeat finders, RECON, RepeatScout and trfinder.

### Gene structure prediction

Trinity^82^ was used to assemble the RNA-Seq data, and the cDNA assembly was used to create gene structures with PASA^83^. The gene structure dataset, with approximately 1,600 complete genes, was used as a training set to train AUGUSTUS^84^ and SNAP^85^. Protein-coding genes in the *P. viticola* genome were predicted independently using three ab initio predictor programs AUGUSTUS, SNAP and GeneMark-ES^86^. Proteomes from five related oomycete species (*H. arabidopsidis, P. sojae, P. ramorum, P. infestans* and *P. ultimum*) and selected UniProt protein sequences from 8 related oomycete species (*P. viticola, H. arabidopsidis, P. sojae, P. ramorum, P. infestans, P. parasitica, P. cubensis and P. ultimum*) were aligned to the *P. viticola* genome using TBLASTN. Proteins with a sequence identity of more than 70% were noted and used as templates to predict the exons of *P. viticola* genes with Genewise^87^. Subsequently, the resultant GFF files from each of the prediction programs were used as inputs into the EVM program^88^ to integrate the data from the three gene prediction programs. Validation of the presence of enzymes related to nitrogen, sulfur and thiamine metabolic pathways was performed by PCR amplification from genomic DNA of ‘JL-7-2’ isolate. PCR amplification was carried out in a 25 μL PrimeStarHS Premix (Takara) containing 10 ng of purified DNA and 0.4 μM of each forward and reverse primer. The reactions were performed in a PCR Labcycler (SENSQUEST, Germany) according to the following program: denaturation was followed by 30 cycles of 10 s at 98 °C, 5 s at 55 °C–58 °C, 1 min/kb at 72 °C, and 5 min of final extension at 72 °C. The PCR product was purified and sequenced by Beijing AuGCT Biological Technology Co., Ltd.

### Metabolic pathway analysis and function annotation

Pathway annotation for *P. viticola* was performed using KAAS (KEGG automated annotation server)^89^. Predicted protein sequences were submitted to KAAS for assigning a KEGG Orthology (KO) identifier. Query sequences were blasted against the KEGG GENES reference database, with homologs selected on the basis of their BLAST score. Homologs were identified as ortholog candidates based on the BLAST score as well as bidirectional best hit information. Ortholog candidates were divided into KO groups according to the annotation of the KEGG GENES database. Finally the assignment score was calculated based on likelihood and heuristics for each KO group. Then, the K ID of the KO group with the highest score was assigned to the query sequence. Once all KO IDs were assigned (essentially the gene products linked to the KEGG pathways), a pathway diagram was constructed. Pathway maps were generated by choosing the non-organism specific option. Function annotation was also analyzed using BLASTP with the NR database. GO and InterPro annotations were performed by Blast2GO^90^.

### Prediction of secreted effectors

Secreted proteins were predicted from the annotated protein set using a local installation of SignalP 3.0 with hidden Markov model methods^91^. TMHMM v2.0^92^ was used to predict transmembrane (TM) domains. Proteins with a predicted signal peptide, but lacking any TM domains (unless overlapping at least 10 amino acids of the signal peptide), were defined as secreted proteins^93^. Potential RXLR and CRN effectors were predicted according to the method of Yin *et al*.^40^.

To identify potential pathogenicity-related genes, BLASTP searches were performed against the Pathogen-Host Interaction database (PHI-base 4.1) with an E-value cut off value of 1e-5^94^. Genes within the *P. viticola* genome encoding putative carbohydrate-active enzymes (CAZymes) were automatically annotated online using the dbCAN database^95^.

### Comparative genomics analysis of sequenced oomycete species

Orthologous pairs were identified using the OrthoMCL program^96^ with an E-value cut-off of 1e-5. Multiple sequence alignments were performed using Mafft^97^. Phylogenetic relationships between different oomycete species were analyzed using Phylip-3.695 using default parameters. Synteny between the genomes of sequenced downy mildews was analyzed using MUMmer v3^98^ and LASTZ^99^.

## Additional Information

**How to cite this article**: Yin, L. *et al*. Genome sequence of *Plasmopara viticola* and insight into the pathogenic mechanism. *Sci. Rep.* **7**, 46553; doi: 10.1038/srep46553 (2017).

**Publisher's note:** Springer Nature remains neutral with regard to jurisdictional claims in published maps and institutional affiliations.

## Supplementary Material

Supplementary Tables and Figures

Supplementary Dataset 5

Supplementary Dataset 7

Supplementary Dataset 9

Supplementary Dataset 10

Supplementary Dataset 12

## Figures and Tables

**Table 1:** *P. viticola* genome assembly statistics and features.

Estimated genome size	101.3 Mb
Number of scaffolds (>500 bp)	2,165.0
Scaffold N50 size (kb)	172.3
Longest scaffold (kb)	805.7
GC content of whole genome (%)	45.0
GC content in exons (%)	49.9
Number of gene models	17,014
Average gene length (bp)	1,344
% Repeats	25.6
Gene density (no. of genes per Mb)	176.0
Mean number of exons per gene	2.2
Mean exon length (bp)	483.0
Mean intron length (bp)	156.4

**Table 2:** Number of orthologous genes between different oomycete pathogen species identified by Ortho MCL.

No. of orthologous genes	*P. viticola*	*P. halstedii*	*H. arabidopsidis*	*P. infestans*	*P. sojae*
*P. viticola*	—	8685	6718	8161	8438
*P. halstedii*	7705	—	6656	8360	8389
*H. arabidopsidis*	6695	7191	—	7469	7539
*P. infestans*	8826	9703	7797	—	13077
*P. sojae*	8974	9824	8145	14420	—
