# Supplementary material for: Genome sequence of Plasmopara viticola and insight into the pathogenic mechanism
Source: Sci Rep. 2017 Apr 18;7:46553. doi: 10.1038/srep46553 (PMC5394536; doi:10.1038/srep46553)

Genome sequence of *Plasmopara viticola* and insight into the pathogenic mechanism

Ling Yin[1,3,¶], Yunhe An[1,2,¶], Junjie Qu[3,¶], Xinlong Li[1], Yali Zhang[1], Ian Dry[5], Huijun Wu[2*], Jiang Lu[1,4]**

[1]College of Food Science and Nutritional Engineering, China Agricultural University, Beijing 100083, China.

[2]Beijing Center for Physical and Chemical Analysis, Beijing 100089, China.

[3]Guangxi Crop Genetic Improvement and Biotechnology Laboratory, Guangxi Academy of Agricultural Sciences, Nanning 530007, China.

[4]Center for Viticulture and Enology, School of Agriculture and Biology, Shanghai Jiao Tong University, Shanghai 200024, China

[5]CSIRO Agriculture& Food, Wine Innovation West Building, Hartley Grove, UrrbraeSA 5064, Australia.

[¶]These authors contributed equally to this work.

[*]Co-corresponding author;

**To whom corresponding should be addressed, E-mail: jiang.lu@sjtu.edu.cn

Supporting information

Supplementary Table S1. Summary of sequencing data information of *P. viticola* isolate ‘JL-7-2’.

Supplementary Table S2. Comparison of *P. viticola* genome assembly statistics using Illumina sequence data only or Illumina/PacBio hybrid assembly.

Supplementary Table S3. Comparison of genome assembly statistics of two sequenced *P. viticola* isolates.

Supplementary Table S4. BUSCO analysis of the genome assembly.

Supplementary Table S5. Transcription of predicted *P. viticola* genes during infection. Number of RNA-Seq reads detected in RNA extracted expressed genes of *P. viticola* isolates ‘JL-7-2’, ‘ZJ-1-1’ and ‘CSIRO-L-2’ during infection of grape leaves. Reads for Chinese isolates ‘JL-7-2’ and ‘ZJ-1-1’ come from RNA-Seq analysis of a mixed RNA sample comprised of equal amounts of total RNA extracted from infected *V. vinifera* cv. Thompson seedless leaves sampled at 24 hpi, 48 hpi and 72 hpi. In contrast, data given for the Australian isolate ‘CSIRO-L-2’ represents the highest number of RNA-Seq reads obtained from RNA sampled at any of the sampling time points of 12 hpi, 24 hpi, 48 hpi, 72 hpi or 96 hpi of *V. vinifera* cv. Cabernet sauvignon leaves. Therefore direct comparisons between the number of reads obtained for each *P. viticola* transcript cannot be made between the Chinese and Australian isolates. Predicted RXLR effectors are highlighted in yellow.

Supplementary Table S6. Summary of repeat elements in the *P. viticola* genome.

Supplementary Table S7. Candidate RXLR/Q effectors encoded in the *P. viticola* genome.

Supplementary Table S8. Gene density and percentage of repeat elements in the *P. viticola* genome and in scaffolds with RXLR clusters.

Supplementary Table S9. Candidate CRN effectors encoded in the *P. viticola* genome.

Supplementary Table S10. Predicted pathogenicity proteins within the *P. viticola* secretome. BLAST analysis was performed with the entire *P. viticola* secretome against the Pathogen-Host Interaction Database (PHI database).

Supplementary Table S11. Putative carbohydrate-active enzyme (CAZyme) classes encoded in the *P.viticola* secretome as predicted using the dbCAN database.

Supplementary Table S12. Differentially expressed genes in *P. viticola* isolate 'ZJ-1-1' during infection of *V. amurensis* cv. Shuanghong.

Supplementary Table S13. Protein identity comparison between all proteins and secreted proteins predicted in *P. viticola* genome.

Supplementary Table S14. Presence and absence of important metabolic enzymes in *P. viticola* and comparision with *Phytophthora* species，other downy mildews and *A. laibachii*. Red indicates absence and green indicates presence of genes. Genes present for all the organisms below were annotated by blast to NCBI non-redundant protein database or KEGG database and genes present in *P. viticola* were also validated by PCR and transcriptome data. The primers were listed in Supplementary Table S15**.**

Supplementary Table S15. Primers were used to validate the presence of enzymes related to nitrogen,

sulfur and thiamine metabolic pathways by PCR amplification from genomic DNA of 'JL-7-2' isolate.

Supplementary Table S1. Summary of sequencing data information of *P. viticola* isolate 'JL-7-2'.

Data Type	Insert Size	Raw reads	Trimmed reads	Sequence Depth
	180 bp	76,363,088	73,311,382	66.1X
Illumina	500 bp	48,061,012	44,068,164	38.6X
Paired-end	800bp	47,564,330	23,866,398	20.4X
	1kb	51,285,508	15,151,256	12.6X
Illumina	3kb	31,320,134	23,352,680	21.0x
Mate pair	6kb	12,799,230	11,471,298	10.2x
PacBio	-	2,404,672	845,976	38.0X
Total	-			207.0X

Supplementary Table S2. Comparison of *P. viticola* genome assembly statistics using Illumina sequence data only or Illumina/PacBio hybrid assembly.

	Illumina Only	Illumina/PacBio assembly
Genome assembly size	103,157,395	101,296,116
Number of ambiguous bp	28,824,422	16,928,280
The percentage of gaps	27.94%	16.71%
N50 of scaffold	160,129	172,266
Length of largest scaffold	692,625	805,677
Total number of scaffolds	2793	2165

Supplementary Table S3. Comparison of genome assembly statistics of two sequenced *P. viticola* isolates.

P.viticola Isolates	JL-7-2 (China, present work)	INRA-PV221 (France[28])
Estimated genome size (without N)	84.37 Mb	72.74 Mb
Number of scaffolds	2,165 (>500 bp)	1,883 (>1000 bp)
Scaffold N50 size (kb)	172.3 kb	180.6 Kb
Longest scaffold (kb)	805.7	763.5
GC content of whole genome (%)	45%	44.3%
% Repeats	25.6%	29.8%
CEGMA/BUSCO (with partials)	97% /90%	95%/na

Supplementary Table S4. Comparison of different oomycete genome assembly by BUSCO analysis. A total of 429 conserved eukaryotes proteins were searched from the assembled *P. viticola* and other 5 oomycete genomes.

Species	Complete	Duplicated	Fragmented	Missing
P. viticola	84%	46%	6%	9.50%
H. arabidopsidis	79%	32%	8.80%	11%
P. halstedii	90%	24%	4.10%	5.30%
P. infestans	90%	29%	2%	6.90%
P. sojae	88%	24%	3.20%	8.30%

Supplementary Table S5. Transcription of predicted *P. viticola* genes during infection. Number of RNA-Seq reads detected in RNA extracted expressed genes of *P. viticola* isolates 'JL-7-2', 'ZJ-1-1' and 'CSIRO-L-2' during infection of grape leaves. Reads for Chinese isolates 'JL-7-2' and 'ZJ-1-1' come from RNA-Seq analysis of a mixed RNA sample comprised of equal amounts of total RNA extracted from infected *V. vinifera* cv. Thompson seedless leaves sampled at 24 hpi,48 hpi and 72 hpi. In contrast, data given for the Australian isolate 'CSIRO-L-2' represents the highest number of RNA-Seq reads obtained from RNA sampled at any of the sampling time points of 12 hpi, 24 hpi, 48 hpi, 72 hpi or 96 hpi of *V. vinifera* cv. Cabernet sauvignon leaves. Therefore direct comparisons between the number of reads obtained for each *P. viticola* transcript cannot be made between the Chinese and Australian isolates. Predicted RXLR effectors are highlighted in yellow.

(See separate excel file)

Supplementary Table S6. Summary of repeat elements in the *P. viticola* genome.

Repeat Elements	Percentage	Number of bases
SINE	0.21%	208618
Unknown	6.55%	6631664
Simple_repeat	0.25%	253996
rRNA	0.08%	79398
RC/Helitron	0.02%	19915
LTR/Gypsy	10.39%	10531330
LTR/Copia	3.15%	3194722
Low_complexity	0.03%	29963
DNA/TcMar-Tc2	-	-
LINE/RTE-X	0.20%	206820
LINE/RTE-BovB	0.33%	336879
LINE/R2	0.02%	23845
LINE/Penelope	0.04%	40107
DNA/TcMar-Pogo	-	-
LTR/Ngaro	-	-
LINE/L1-Tx1	1.08%	1096284
LINE/L1	0.28%	283826
LINE/R1	-	-
LINE/telomeric	-	-
DNA/TcMar-Tc1	-	-
LINE/Jockey	0.11%	115846
DNA/Harbinger	-	-
DNA/TcMar-Tigger	0.03%	32269
DNA/TcMar-Fot1	0.07%	74427
DNA/TcMar-	-	-
DNA/Sola	0.14%	137774
DNA/MuLE-MuDR	0.68%	690357
DNA/Maverick	1.61%	1633176
DNA/hAT-hobo	0.02%	18627
DNA/hAT-Ac	0.09%	93893
DNA/hAT-Charlie	-	-
DNA/hAT-Tag1	-	-
DNA/hAT-Tip100	-	-
DNA/Crypton	0.15%	149261
Total	25.6 %	25882997

Supplementary Table S7. Candidate RXLR/Q effectors encoded in the *P. viticola* genome.

(See separate excel file)

Supplementary Table S8. Gene density and percentage of repeat elements in the *P. viticola* genome and in scaffolds with RXLR clusters.

	Gene Density (# gene per Mb)	Percentage of repeat elements
Seven scaffolds containing RXLR clusters	139	36.09%
All *P. viticola* genome scaffolds	176	25.55%

Supplementary Table S9. Candidate CRN effectors in the *P. viticola* genome.

(See separate excel file)

Supplementary Table S10. Predicted pathogenicity proteins within the *P. viticola* secretome. BLAST analysis was performed with the entire *P. viticola* secretome against the Pathogen-Host Interaction Database (PHI database).

(See separate excel file)

Supplementary Table S11. Putative carbohydrate-active enzyme (CAZyme) classes encoded in the *P. viticola* secretome as predicted using the dbCAN database.

	CAZy families	Number	Total number
**Auxiliary Activities (AAs)**	AA10	1	4
	AA7	1	
	AA7	1	
	AA8	1	
**Carbohydrate-Binding Modules (CBMs)**	CBM1	4	9
	CBM13	1	
	CBM15	1	
	CBM50	1	
	CBM63	1	
	CBM9	1	
**Carbohydrate Esterases (CEs)**	CE1	4	16
	CE10	2	
	CE13	1	
	CE4	2	
	CE5	1	
	CE8	6	
**Glycoside Hydrolases(GHs)**	GH102	1	60
	GH109	1	
	GH131	13	
	GH17	9	
	GH19	3	
	GH23	2	
	GH28	1	
	GH3	5	
	GH30	1	
	GH32	2	
	GH43	1	
	GH5	3	
	GH6	13	
	GH7	1	
	GH72	4	
**Glycosyltransferases (GTs)**	GT31	4	7
	GT4	2	
	GT71	1	
**Polysaccharide lyases (PLs)**	PL22	1	1

Supplementary Table S12. Differentially expressed genes in *P. viticola* isolate ‘ZJ-1-1’ during infection of *V. amurensis* cv. Shuanghong.

(See separate excel file)

Supplementary Table S13. Amino acid identity comparison between all proteins and secreted proteins predicted in *P. viticola* compared to other ooymecete genomes.

>60%amino acid identity	*P. halstedii*	*P. infestans*	*P. sojae*	*H. arabidopsidis*
All proteins (*P. viticola*)	9657 (56.8%)	8545 (50.2%)	8164(50.0%)	6035 (35.5%)
Secreted proteins (*P. viticola*)	733 (43.5%)	698 (41.4%)	672 (39.9%)	426 (25.3%)

Supplementary Table S14. Presence and absence of important metabolic enzymes in *P. viticola* and comparision with *Phytophthora* species, other downy mildews and *A. laibachii*. Red indicates absence and green indicates presence of genes. Genes present for all the organisms below were annotated by blast to NCBI non-redundant protein database or KEGG database and genes present in *P. viticola* were also validated by PCR and transcriptome data. The primers were listed in Supplementary Table S14**.**

	P. viticola	*P. halstedii*[1]	*H. arabidopsidis*[2]	*P. sojae*[2]	*P. infestans*[2]	*A.laibachii* [3]
Nitrate reductase	Pv11323	Phal04323	none	Ps140563	PITG_13012.1	none
Nitrite reductase	none	none	none	Ps140562	PITG_13013.1	none
Nitrate transporter	Pv07654	Phal07021	none	Ps140564	PITG_13011.1	CCA24412
Sulphite oxidase	Pv02147	Phal16715	Ha812582	Ps250369	PITG_12342.1	none
	Pv10357	Phal03654				
Sulphite reductase	none	none	none	Ps139488	PITG_19263.1	none
				Ps139493	PITG_18187.1	
Cysteine synthetase	Pv02771	Phal08105	Ha814750	Ps109172	PITG_12727.1	none
	Pv05868			Ps109175	PITG_12725.1	
Thiamine-phosphate synthase	none	none	none	none	none	none
Thiamine pyrophosphokinase	Pv08978	Phal05670	Ha803592	Ps565547	PITG_07866.1	CCA24794
	Pv10004					

1Gene IDs from the Broad Institute Database: http://www.broadinstitute.org.

2 Gene IDs from the VBI Microbial Database: vmd.vbi.vt.edu.

3 Gene IDs fromhttp://dataportal-senckenberg.de/database/metacat/rsharma.26.2/bikf.

Supplementary Table S15. Primers were used to validate the presence of enzymes related to nitrogen, sulfur and thiamine metabolic pathways by PCR amplification from genomic DNA of 'JL-7-2' isolate.

Primers	Sequences (5'to3')
Pv11323-F	GTGGAACAAGAGTGGCTGGATACGG
Pv11323-R	GTCCAAGACACTCCAGCA
Pv07654-F	ATGCCACAAAGTGCATCGTAC
Pv07654-R	TTACTCGATAGGCGCTAGTTG
Pv08978-F	TGTTATCGGACGACACTA
Pv08978-R	GATGCCACCACTTCTACT
Pv10004-F	GTTACTAAAGAGGGCGAAAG
Pv10004-R	AGACGCAGTGAGTCCAAAT

Figure S1. Genomic distribution of contig length (N length) versus contig number (N number). N lengths were calculated by ordering all sequences according to their length and then adding the length from longest to shortest until the summed length exceeded 10% (N10), 20% (N20), etc., up to 100% (N100) of the assembled contigs. Plotting the N length versus the N number (number of contigs in each N category) indicates that 90% of the assembled genome show high continuity, while the last 10% are highly fragmented.

Figure S2. Identification of single copy core eukaryotic orthologous genes (CEGs) by the CEGMA pipeline. Group 1 represents the least conserved of the 248 core eukaryotic genes, with the degree of conservation increasing in subsequent groups through Group 4.

Figure S3. Highly conserved RXLR effectors identified in multiple oomycete species. Multiple sequence alignment of two conserved PvRXLRs (a) and one PvCRN (b) between different oomycete species. The RXLR and dEER motifs are indicated with a black boxes. The alignment was constructed using BioEdit3.3.19.0 software. The threshold (%) for shading was set at 50. Similar amino acid residues are shaded grey and identical amino acid residues are shaded black.

Figure S4. Molecular divergence (a) and phylogenetic relationship (b) between *P. viticola* and other species based on pairwise comparisons of the one-to-one orthologues. In the figure (a), the cumulative frequencies of amino acid identity across each set of potential orthologous pairs is presented.

Figure S1.

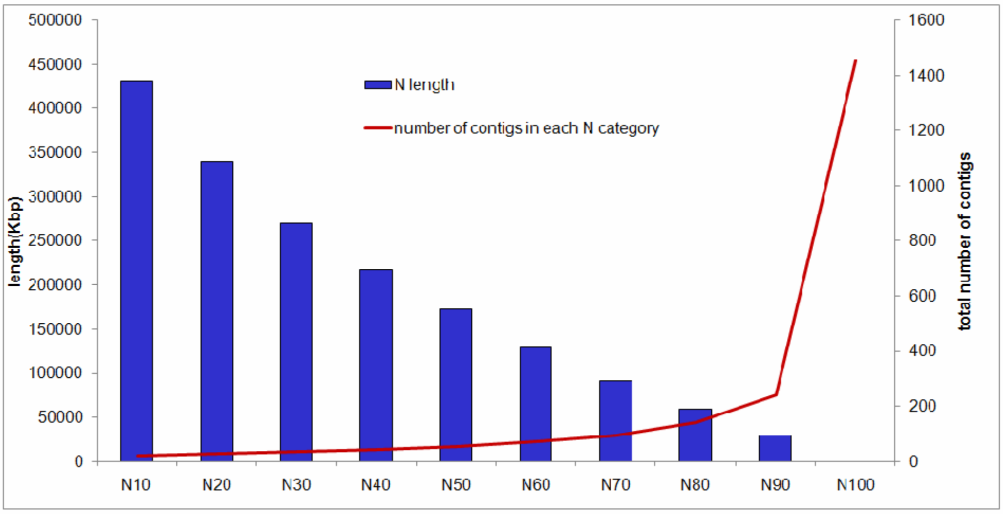

Figure S2.

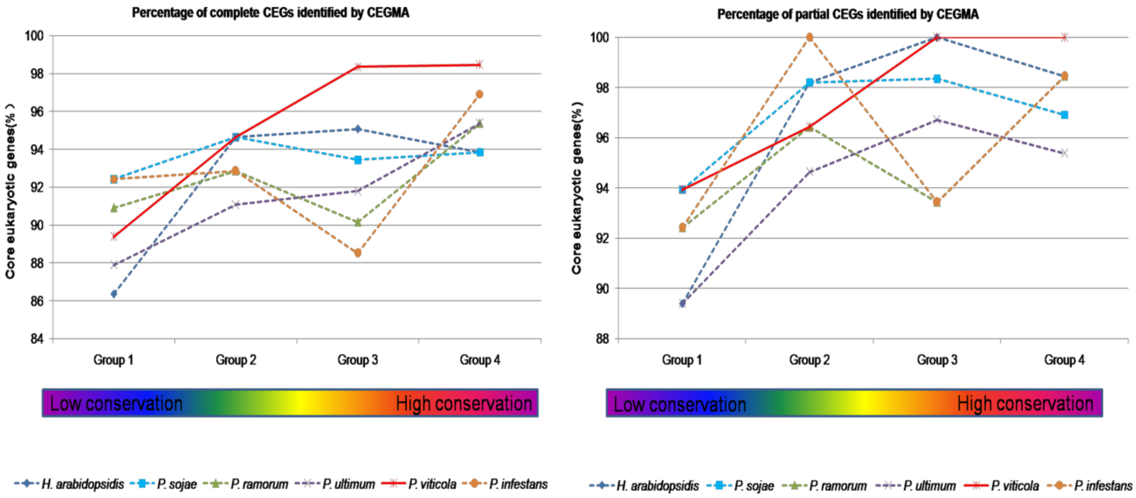

Figure S3.

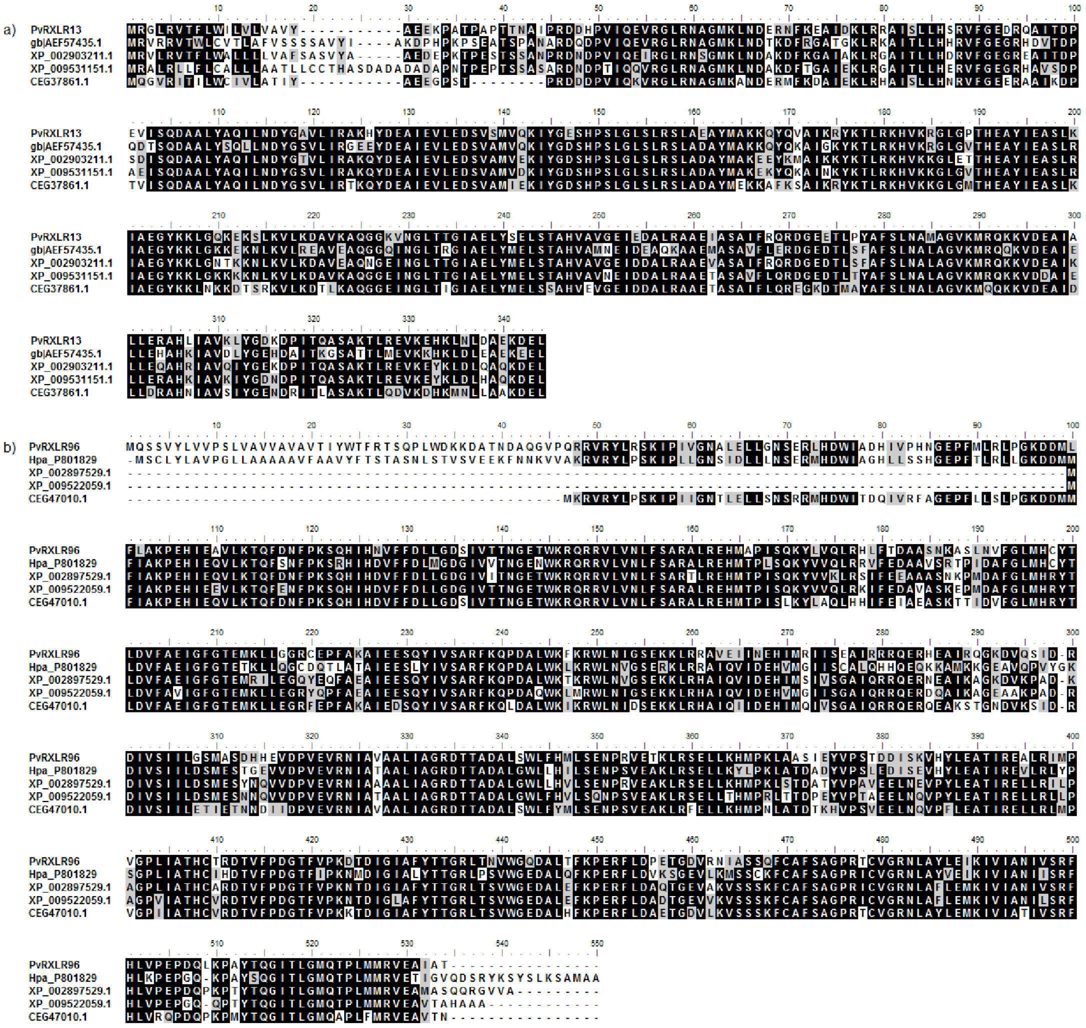

Figure S4.

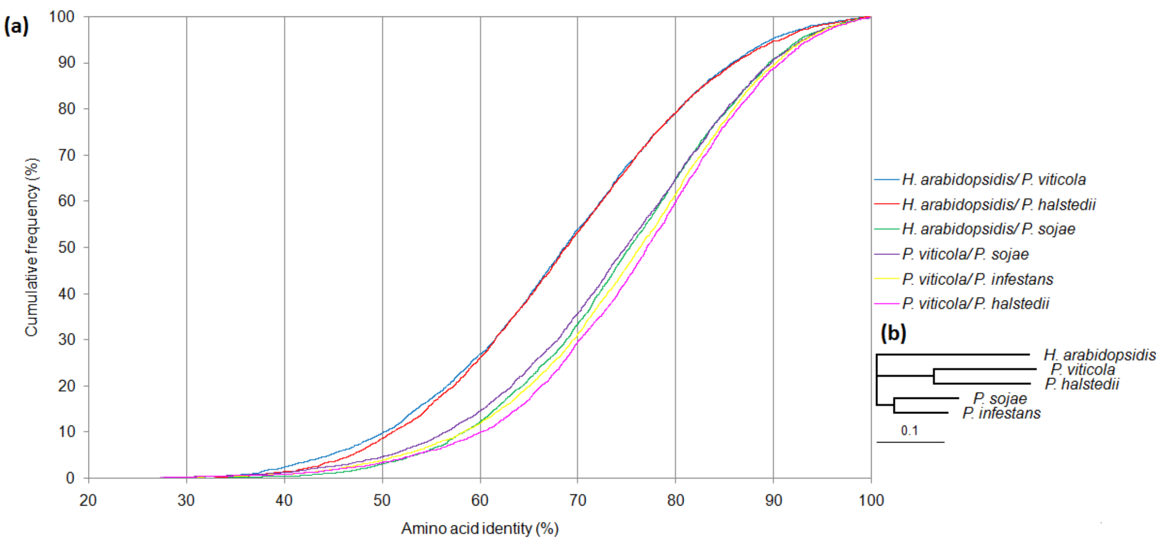

Supplement: Supplementary Tables and Figures [file srep46553-s1.pdf]
